# The impact of proton pump inhibitor exposure on pneumonia: an updated meta-analysis based on randomized controlled trials

**DOI:** 10.3389/fphar.2025.1713256

**Published:** 2025-10-28

**Authors:** Zhujun Wu, Yixuan Wu, Zhiyi Xiang, Yi Qiu, Wangyi Xuan, Shengying Zhang

**Affiliations:** ^1^ Department of Gastroenterology, Ningbo Zhenhai Hospital of Traditional Chinese Medicine, Ningbo, Zhejiang, China; ^2^ The Second Clinical Medical College, Zhejiang Chinese Medical University, Hangzhou, Zhejiang, China; ^3^ The First Clinical Medical College, Zhejiang Chinese Medical University, Hangzhou, Zhejiang, China; ^4^ Department of Respiratory and Critical Care Medicine, Ningbo Yinzhou No. 2 Hospital, , Ningbo, Zhejiang, China

**Keywords:** proton pump inhibitor, pneumonia, asia, pantoprazole, hospital-acquired pneumonia, meta-analysis

## Abstract

**Background:**

The association between proton pump inhibitor (PPI) use and pneumonia risk remains inconclusive. This meta-analysis explores the impact of PPI use on the risk of pneumonia.

**Methods:**

We systematically searched for relevant randomized controlled trials in PubMed, Web of Science, EMBASE and Cochrane Library from January 2000 to March 2025. Relative ratio and 95% confidence interval were calculated to quantify the association between proton pump inhibitor use and pneumonia incidence.

**Results:**

The analysis included 20 RCTs involving 29,100 participants. Compared to the non-PPI group, the PPI-exposed group showed a higher incidence of pneumonia in the general population (RR = 1.10, 95% CI: 0.99-1.21, p = 0.07) and for hospital-acquired pneumonia (HAP) (RR = 1.12, 95% CI: 1.00-1.26, p = 0.06), although the differences were not statistically significant. A higher incidence of pneumonia was observed in the intervention group among the Asian population (RR = 1.30, p = 0.02), particularly in Iran (RR = 2.73, p < 0.001) and among Asian users of pantoprazole (RR = 1.94, p = 0.05). No significant differences in pneumonia incidence were found between groups among participants from Europe (RR = 1.04, p = 0.67) or America (RR = 1.02, p = 0.95), for ventilator-associated pneumonia (VAP) (RR = 1.17, p = 0.11), or among participants in intensive care units (ICUs) (RR = 1.05, p = 0.29) or out of ICUs (RR = 1.28, p = 0.14).

**Conclusion:**

The use of PPI might increase the risk of pneumonia in general population, especially among Asians (in Iran and in the Asian users of pantoprazole), HAP.

**Systematic Review Registration:**

Identifier, CRD420251021884.

## Introduction

Thirty years after its clinical introduction, the proton pump inhibitor (PPI), renowned for its potent gastric acid suppression, is widely recommended as first-line treatment for acid-related disorders such as peptic ulcer disease, gastroesophageal reflux disease, erosive esophagitis (Scarpignato et al., 2016), and Zollinger-Ellison syndrome. PPIs are also effective in *Helicobacter pylori* eradication regimens (Li et al., 2020) and for gastric protection in patients using non-steroidal anti-inflammatory drugs (NSAIDs) (Garegnani et al., 2025) or antiplatelet therapy (Saeed et al., 2025).

However, PPI prescriptions frequently exceed guideline recommendations for indications and duration (Shanika et al., 2023), leading to increasing concerns about potential health hazards and unnecessary economic costs. Factors contributing to this overuse include the easy availability and cost-effectiveness of PPIs, coupled with insufficient awareness among both the public and healthcare providers regarding evidence for PPI discontinuation (Heidelbaugh et al., 2012). An observational study using a Dutch primary care database revealed that over half of PPI users in primary care lacked appropriate indications, particularly for unnecessary ulcer prophylaxis related to concomitant medication use (Koggel et al., 2022). Globally, it is estimated that at least two billion pounds are wasted annually on unnecessary PPI prescriptions (Forgacs and Loganayagam, 2008).

Although PPIs are generally considered safe and well-tolerated, accumulating evidence suggests potential adverse effects. A review from British Columbia summarized PPI-associated risks, including *Clostridium difficile* and other enteric infections, cardiovascular events, acute kidney injury, gastrointestinal tumors, and osteoporotic fractures (Ben-Eltriki et al., 2024). A Taiwanese cohort study found that PPI use in patients with non-traumatic intracranial hemorrhage was associated with an increased risk of pneumonia (Ho et al., 2014). While current meta-analyses have investigated the correlation between PPI utilization and pneumonia incidence (Xun et al., 2022; Wang et al., 2024a), the persistent debates regarding the results, along with the progressive developments in clinical trials, underscore the imperative for a thorough risk assessment of pneumonia in relation to PPI therapy. Given these considerations, we conducted an updated meta-analysis of randomized controlled trials (RCTs) with comprehensive subgroup analyses to investigate the influence of PPI use on pneumonia risk.

## Methods

### Study registration

Adhering to the Preferred Reporting Items for Systematic Reviews and Meta-Analyses (PRISMA) guidelines (Page et al., 2021), this meta-analysis was registered with the International Prospective Register of Systematic Reviews (PROSPERO) with the registration number CRD420251021884. A systematic search was conducted across four databases (PubMed, Web of Science, Embase, and Cochrane Library) from January 2000 to March 2025. The search strategy utilized the following keywords: ((Proton pump inhibitor) OR PPI OR omeprazole OR pantoprazole OR rabeprazole OR esomeprazole OR lansoprazole OR dexlansoprazole OR ilaprazole OR tenatoprazole) AND (pneumonia OR (ventilator-associated pneumonia) OR VAP OR (pulmonary infection OR lung inflammation) OR (pulmonary inflammation)). Additionally, reference lists of included studies were manually screened to identify potentially relevant publications not captured by the database search.

### Selection and exclusion criteria

Studies were selected based on the PICOS (Participants, Intervention, Comparison, Outcomes, Study design) framework (Rudwaleit et al., 2009). The inclusion criteria were as follows: (1) participants: patients without pre-existing pneumonia; (2) intervention: intravenous or oral (including percutaneous endoscopic gastrostomy [PEG] or nasogastric [NG] tube) PPI administration; (3) comparison: no drug, matching placebo, H2 receptor antagonist, sucralfate, or gefarnate; (4) outcomes: Incidence of pneumonia (pneumonia was considered to be ventilator-associated pneumonia [VAP] if it occurred after a minimum of 48 h after the initiation of mechanical ventilation and pneumonia was considered to be hospital-acquired pneumonia [HAP] if it occurred during hospitalization); (5) study design: only RCTs.

Exclusion criteria were: (1) studies without full-text availability; (2) studies published in languages other than English; (3) studies lacking accessible data or data unsuitable for meta-analysis; (4) duplicate publications (the most recent or complete version was retained).

### Data extraction and quality assessment

Two independent investigators extracted data using a predefined form, capturing study characteristics (authors, publication year, country, recruitment year, therapeutic regimen), participant characteristics (the number of patients, comorbidities), and the type of pneumonia. If a single study contained multiple independent arms, each arm was treated as a separate data entry in the meta-analysis to avoid unit-of-analysis error and to utilize all available evidence.

For RCTs, the Cochrane Collaboration Network (Higgins et al., 2011) in Revman version 5.4 was used to assess the methodological quality of included RCTs across seven domains. (1) random sequence generation (selection bias), (2) allocation concealment (selection bias), (3) blinding of participants and personnel (performance bias), (4) blinding of outcome assessment (detection bias), (5) incomplete outcome data (attrition bias), (6) selective reporting (reporting bias), (7) other bias. Each study was independently rated as “low risk”, “high risk”, or “unclear risk” for each domain by two authors. Disagreements were finally resolved after discussion.

### Statistical analysis

Statistical analyses were performed using RevMan 5.4. The effect of PPI use on pneumonia risk was estimated using relative risks (RRs) with 95% confidence intervals (CIs). An RR > 1 indicates that the control group is supported while an RR < 1 reveals the result favors the experimental group. Heterogeneity was assessed using the chi-square test and quantified by the *I* ^*2*^ statistic (*I* ^*2*^ < 25%, low; 25%–50%, moderate; >50%, substantial heterogeneity) (Higgins et al., 2003). Due to anticipated clinical and methodological diversity among studies, a random-effects model was employed for all analyses to enhance result robustness. Subgroup analyses were performed to explore heterogeneity sources. Publication bias was evaluated using funnel plots and Begg’s test. Sensitivity analyses assessed result stability by sequentially excluding individual studies. Statistical significance was defined as a two-sided p-value <0.05.

## Results

### Study selection

**FIGURE 1:**
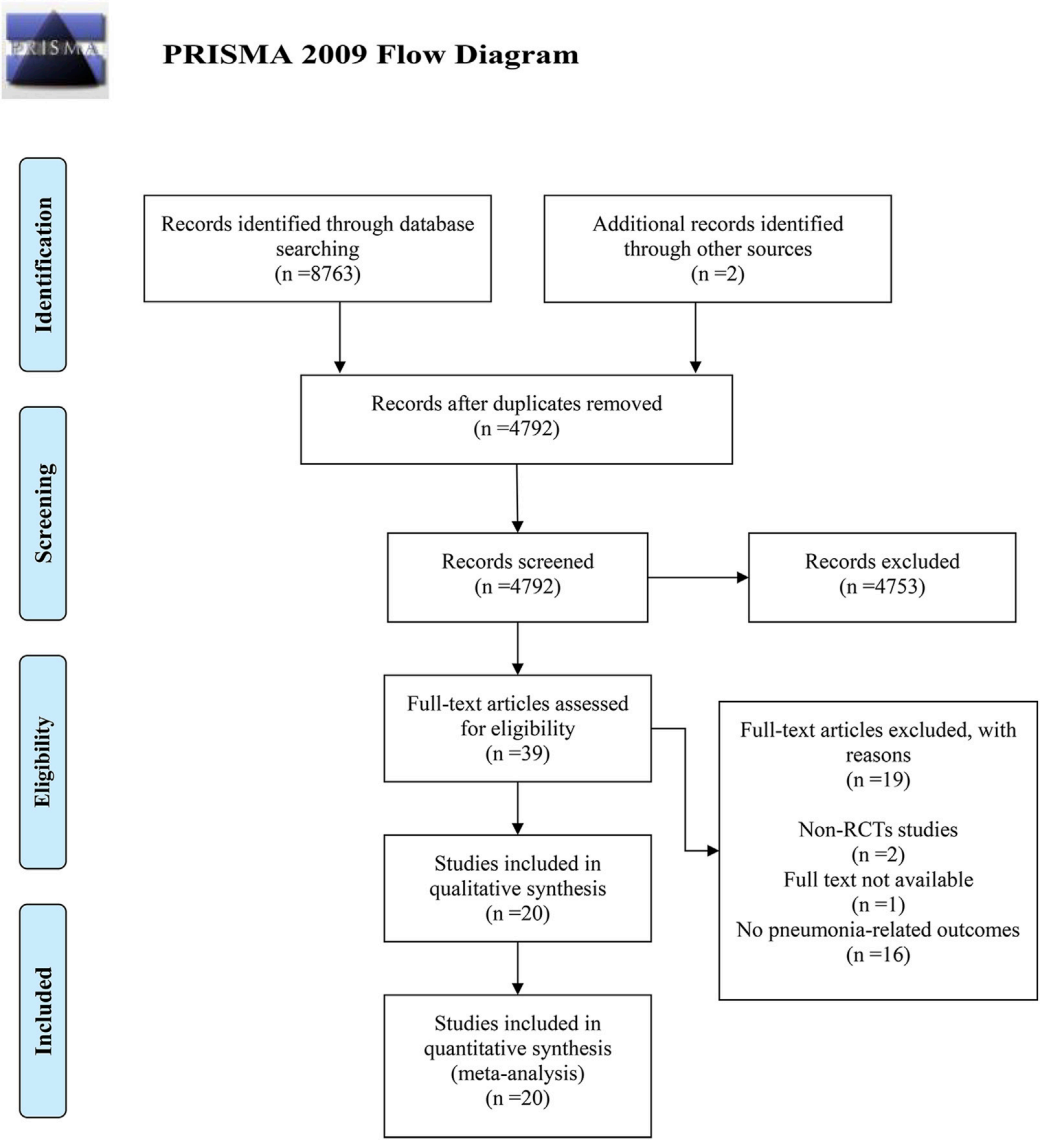
Flow chart of study selection.

Literature searches were executed across four databases using specific search patterns, yielding a combined total of 8,763 records. Manual searches of reference lists identified two additional records. After duplicate removal, 4,792 unique records remained. Screening of titles and abstracts excluded 4,753 records deemed irrelevant, leaving 39 potentially relevant full-text articles for eligibility assessment. Of these, 19 articles were excluded: two were non-RCTs, one had unobtainable full text, and 16 did not report pneumonia-related outcomes. Ultimately, 20 RCTs meeting the inclusion criteria were included in the meta-analysis. Further details of the retrieval process are presented in Figure 1.

### Characteristics and quality assessment of included studies

Twenty RCTs (Abu El-Ella et al., 2022; Alhazzani et al., 2017; Bashar et al., 2013; Cook et al., 2024; Holbrook et al., 2012; Khorvash et al., 2014; Krag et al., 2018; Lee et al., 2014; Lin et al., 2016; Liu et al., 2013; Lou et al., 2018; Moayyedi et al., 2019; Oliynyk, 2021; Orenstein et al., 2009; Selvanderan et al., 2016; Somberg et al., 2008; Sugano et al., 2012; Takatori et al., 2013; Wu et al., 2011; Yildizdas et al., 2002) published between 2002 and 2024 investigated the association between PPI use and pneumonia-related adverse outcomes, involving 14,567 participants in the intervention group and 14,533 in the control group. In the intervention group, pantoprazole was used in 9 studies (Alhazzani et al., 2017; Bashar et al., 2013; Cook et al., 2024; Khorvash et al., 2014; Krag et al., 2018; Moayyedi et al., 2019; Selvanderan et al., 2016; Somberg et al., 2008; Wu et al., 2011), lansoprazole in 5 (Holbrook et al., 2012; Lin et al., 2016; Orenstein et al., 2009; Sugano et al., 2012; Takatori et al., 2013), omeprazole in 4 (Abu El-Ella et al., 2022; Liu et al., 2013; Oliynyk, 2021; Yildizdas et al., 2002), and esomeprazole in the remaining 2 (Lee et al., 2014; Lou et al., 2018). Control groups received: no drugs or placebo (11 studies) (Abu El-Ella et al., 2022; Alhazzani et al., 2017; Cook et al., 2024; Holbrook et al., 2012; Krag et al., 2018; Lin et al., 2016; Moayyedi et al., 2019; Oliynyk, 2021; Orenstein et al., 2009; Selvanderan et al., 2016; Wu et al., 2011), H2 receptor antagonists (4 studies) (Bashar et al., 2013; Lee et al., 2014; Lou et al., 2018; Somberg et al., 2008), sucralfate (1 study) (Khorvash et al., 2014), gefarnate (1 study) (Sugano et al., 2012), placebo plus cimetidine (1 study) (Liu et al., 2013), no drugs plus mosapride (1 study) (Takatori et al., 2013), or were stratified into sucralfate, ranitidine, and no-drug groups (1 study) (Yildizdas et al., 2002). Adverse outcomes were reported as VAP in 11 RCTs (Abu El-Ella et al., 2022; Alhazzani et al., 2017; Bashar et al., 2013; Cook et al., 2024; Khorvash et al., 2014; Lee et al., 2014; Lin et al., 2016; Lou et al., 2018; Oliynyk, 2021; Selvanderan et al., 2016; Yildizdas et al., 2002) and HAP in 16 RCTs (Abu El-Ella et al., 2022; Alhazzani et al., 2017; Bashar et al., 2013; Cook et al., 2024; Khorvash et al., 2014; Krag et al., 2018; Lee et al., 2014; Lin et al., 2016; Liu et al., 2013; Lou et al., 2018; Oliynyk, 2021; Selvanderan et al., 2016; Somberg et al., 2008; Takatori et al., 2013; Wu et al., 2011; Yildizdas et al., 2002). In the remaining 4 RCTs (Holbrook et al., 2012; Moayyedi et al., 2019; Orenstein et al., 2009; Sugano et al., 2012), no details were provided about specific VAP or HAP cases. Regarding comorbidities, 3 RCTs (Lee et al., 2014; Liu et al., 2013; Oliynyk, 2021) enrolled participants with neurological injury and 2 (Moayyedi et al., 2019; Wu et al., 2011) recruited patients with cardiovascular disease (CVD). Fourteen RCTs (Abu El-Ella et al., 2022; Alhazzani et al., 2017; Bashar et al., 2013; Cook et al., 2024; Khorvash et al., 2014; Krag et al., 2018; Lee et al., 2014; Lin et al., 2016; Liu et al., 2013; Lou et al., 2018; Oliynyk, 2021; Selvanderan et al., 2016; Somberg et al., 2008; Yildizdas et al., 2002) included participants in intensive care units (ICUs), while 6 (Holbrook et al., 2012; Moayyedi et al., 2019; Orenstein et al., 2009; Sugano et al., 2012; Takatori et al., 2013; Wu et al., 2011) focused on participants in general wards or non-hospital settings. Further details are presented in Table 1 and Table 2.

**TABLE 1:** Characteristics of all the studies included in the meta-analysis.

Author, year	Country	Recruitment year	Intervene		Control		Age	Type of pneumonia
			Type of drug	No. of participant	Type of drug	No. of participant		
Abu El-Ella et al. (2022)	Egypt	2019–2020	omeprazole	72	no	72	1 month-16 years	HAP, VAP
Alhazzani et al. (2017)	Multicenter	2015	pantoprazole	49	placebo	42	≥18 years	VAP
Bashar et al. (2013)	Iran	2011–2012	pantoprazole	60	ranitidine	60	≥18 years	VAP
Cook et al. (2024)	Multicenter	2019–2023	pantoprazole	2394	placebo	2381	≥18 years	VAP
Holbrook et al. (2012)	America	2007–2011	lansoprazole	147	placebo	150	6 years-17 years	/
Khorvash et al. (2014)	Iran	2010–2011	pantoprazole	66	sucralfate	71	10 years-89 years	VAP
Krag et al. (2018)	Multicenter	2016–2017	pantoprazole	1644	placebo	1647	≥18 years	HAP
Lee et al. (2014)	China	2007–2010	esomeprazole	30	famotidine	30	≥18 years	VAP
Lin et al. (2016)	China	2009–2012	lansoprazole	60	no	60	≥18 years	VAP
Liu et al. (2013)	China	2006–2008	omeprazole	58	cimetidine	54	>18 years	HAP
					placebo	53		
Lou et al. (2018)	China	2014–2016	esomeprazole	147	cimetidine	153	18 years- 70 years	VAP
Moayyedi et al. (2019)	Multicenter	2013–2016	pantoprazole	8791	placebo	8807	≥65 years	/
Oliynyk (2021)	Ukraine	2018–2019	omeprazole	100	placebo	100	/	VAP
Orenstein et al. (2009)	America, Poland	2006–2007	lansoprazole	81	Placebo	81	28 days-12 months	/
Selvanderan et al. (2016)	Australia	2014–2015	pantoprazole	106	placebo	108	≥18 years	VAP
Somberg et al. (2008)	America	2000–2001	pantoprazole	167	cimetidine	35	≥18 years	HAP
Sugano et al. (2012)	Japan	/	lansoprazole	183	gefarnate	181	25 years- 85 years	/
Takatori et al. (2013)	Japan	2009–2011	lansoprazole	41	no	38	≥48 years	AP
					mosapride	40		
Wu et al. (2011)	China	2008–2010	pantoprazole	333	placebo	332	>18 years	HAP
Yildizdas et al. (2002)	Turkey	2000–2002	omeprazole	38	sucralfate	38	<18 years	VAP
					ranitidine	42		
					no	42		

No, number; mo, month; y, year; d, day; wk, week; HAP, hospital-acquired pneumonia; VAP, ventilator-associated pneumonia; AP, aspiration pneumonia.

**TABLE 2:** Characteristics of all the studies included in the meta-analysis.

Author, year	Clinical trial number	Comorbidity
Abu El-Ella et al. (2022)	/	patients with mild to moderate organ dysfunction in PICU
Alhazzani et al. (2017)	NCT02290327	Patients were undergoing invasive mechanical ventilation in ICU
Bashar et al. (2013)	/	Trauma patients were undergoing invasive mechanical ventilation in ICU
Cook et al. (2024)	NCT03374800	Patients were undergoing invasive mechanical ventilation in ICU
Holbrook et al. (2012)	NCT00442013	Children with poor asthma control without symptomatic gastroesophageal reflux
Khorvash et al. (2014)	/	Patients were undergoing invasive mechanical ventilation in ICU
Krag et al. (2018)	NCT02467621	Patients were admitted to the ICU for an acute condition and had at least one risk factor for clinically important gastrointestinal bleeding
Lee et al. (2014)	NCT00633035	patients were admitted to the neurosurgical ICU for post-surgical care or management of severe cerebrovascular accident
Lin et al. (2016)	NCT00708149	Patients were admitted to the respiratory care center due to difficulties being weaned off ventilators in the medical or surgical ICUs
Liu et al. (2013)	ChiCTR-TRC-12001871	Patients had CT-proven ICH within 72 h of ictus requiring neurosurgery in neurosurgical ICU
Lou et al. (2018)	NCT02157376	Patients in ICU with an anticipated stay of at least 72 h, were expected to survive for at least 48 h, and required a mechanical ventilator for an anticipated minimum of 48 h, and had at least one additional risk factor for stress-ulcer bleeding
Moayyedi et al. (2019)	NCT01776424	Patients with stable atherosclerotic vascular disease and were using anti-coagulation strategies
Oliynyk (2021)	0119U002307	Patients with severe craniocerebral injury that underwent surgery for this pathology and subsequently developed sepsis in the postoperative period
Orenstein et al. (2009)	NCT00324974	Infants with symptoms attributed to gastroesophageal reflux disease that have persisted despite a >1 week course of nonpharmacologic management
Selvanderan et al. (2016)	ACTRN12613000807752	Patients who were anticipated to be invasively mechanically ventilated for greater than 24 h and receive enteral nutrition within 48 h
Somberg et al. (2008)	/	Patients in ICU with at least one of the risk factors for stress-related UGI bleeding
Sugano et al. (2012)	NCT00787254	Patients with gastric or duodenal ulcers associated with long-term NSAID therapy excluding low-dose aspirin
Takatori et al. (2013)	/	Patients being fed with liquid nutrients via a PEG tube
Wu et al. (2011)	/	patients with ACS who are at high risk for GI bleeding
Yildizdas et al. (2002)	/	patients who needed mechanical ventilation in PICU

PICU, pediatric intensive care unit; ICU, intensive care unit; ICH, intracranial hemorrhage; UGI, upper gastrointestinal; GI, gastrointestinal; NSAID, nonsteroidal anti-inflammatory drug; PEG, percutaneous endoscopic gastrostomy; ACS, acute coronary syndromes.

Risk of bias was assessed for all included studies using the Cochrane Collaboration tool across seven domains (Supplementary Figure S1; Supplementary Figure S2). Three open-label trials were rated “high risk” for both performance bias and detection bias. Two studies demonstrated high risk of performance bias due to unblinding of participants. Overall, all RCTs were judged to be higher-quality studies.

### Analysis of the primary result

**FIGURE 2:**
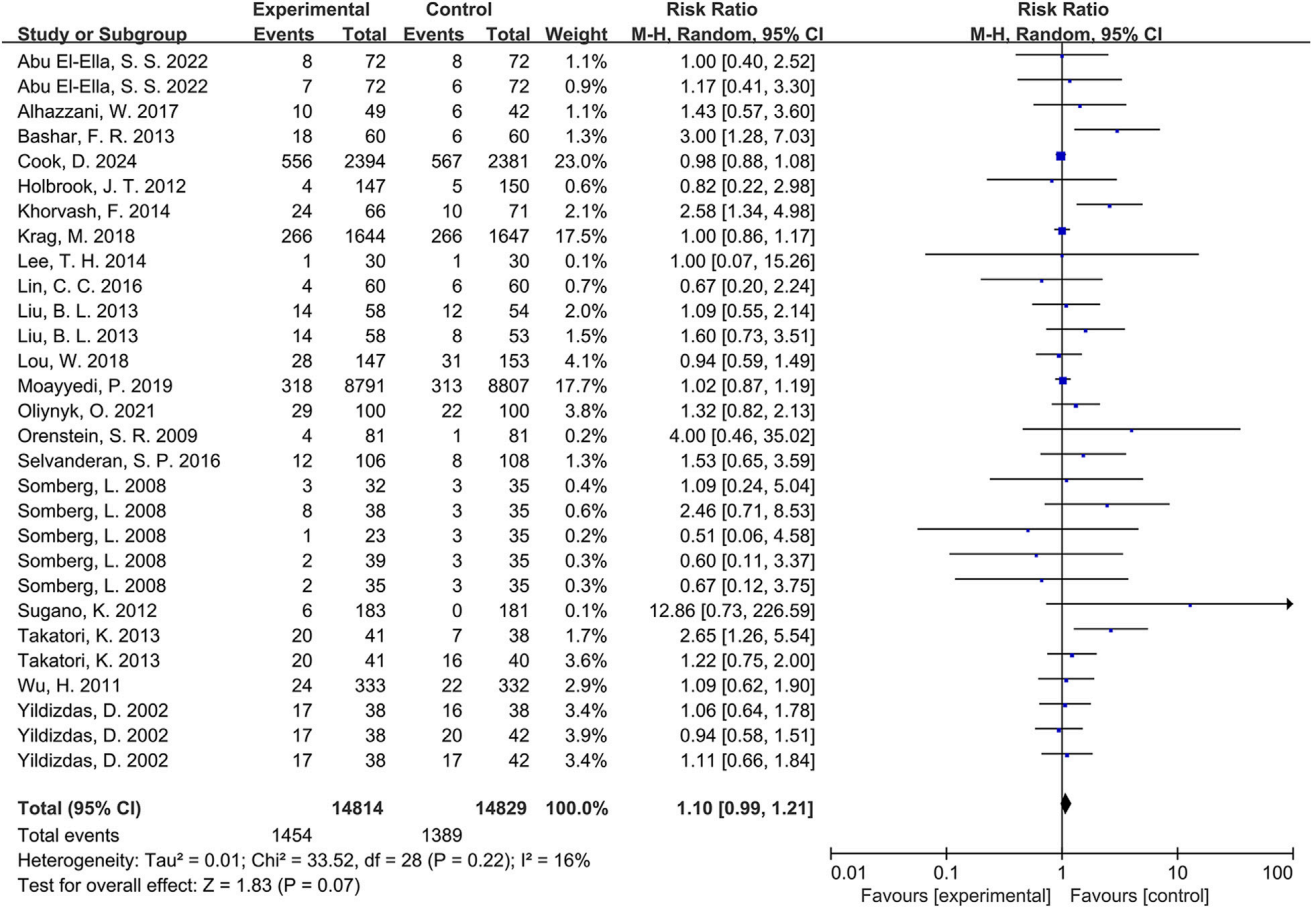
Forest plot of the effect of proton pump inhibitor use on the incidence of pneumonia.

Meta-analysis of pneumonia outcomes across the 20 included RCTs (Abu El-Ella et al., 2022; Alhazzani et al., 2017; Bashar et al., 2013; Cook et al., 2024; Holbrook et al., 2012; Khorvash et al., 2014; Krag et al., 2018; Lee et al., 2014; Lin et al., 2016; Liu et al., 2013; Lou et al., 2018; Moayyedi et al., 2019; Oliynyk, 2021; Orenstein et al., 2009; Selvanderan et al., 2016; Somberg et al., 2008; Sugano et al., 2012; Takatori et al., 2013; Wu et al., 2011; Yildizdas et al., 2002) demonstrated a higher incidence in PPI-exposed groups compared to controls. However, this difference did not reach statistical significance (RR = 1.10, 95% CI: 0.99-1.21, p = 0.07; Figure 2). Furthermore, low heterogeneity was observed among the studies (*I* ^*2*^ = 16%).

### Subgroup analysis

Age-stratified subgroup analyses revealed no significant differences in pneumonia incidence between intervention and control groups for either the <18-year group (RR = 1.04, p = 0.74) or the ≥18-year group (RR = 1.07, p = 0.23). Additionally, analysis of two studies (Moayyedi et al., 2019; Takatori et al., 2013) exclusively involving older adults demonstrated no between-group difference in pneumonia rates (RR = 1.32, p = 0.23). Comprehensive subgroup data are presented in Table 3.

**TABLE 3:** Subgroup analysis of the risk of pneumonia.

Subgroup	No. of studies	RR (95% CI)	P	I^2^
Age				
<18 years	4	1.04 [0.81, 1.35]	0.74	0%
≥18 years	14	1.07 [0.96, 1.19]	0.23	18%
Older adults	2	1.32 [0.84, 2.00]	0.23	69%
Comorbidity				
Nerve injury	3	1.30 [0.92, 1.83]	0.14	0%
CVD	2	1.02 [0.88, 1.19]	0.77	0%
ICU				
Yes	14	1.05 [0.96, 1.16]	0.29	5%
No	6	1.28 [0.92, 1.79]	0.14	45%
Country				
Asia	10	1.30 [1.04, 1.64]	0.02	38%
Europe	2	1.04 [0.86, 1.26]	0.67	12%
America	2	1.02 [0.54, 1.91]	0.95	0%
Type of pneumonia				
VAP	11	1.17 [0.97, 1.41]	0.11	33%
HAP	16	1.12 [1.00, 1.26]	0.06	17%
Type of PPI				
Omeprazole	4	1.12 [0.91, 1.39]	0.28	0%
Lansoprazole	5	1.52 [0.84, 2.74]	0.16	43%
Pantoprazole	9	1.09 [0.95, 1.26]	0.21	37%
Esomeprazole	2	0.95 [0.60, 1.48]	0.79	0%
Type of control group				
Placebo or no drug	14	1.01 [0.95, 1.09]	0.69	0%
H2 receptor antagonist	6	1.10 [0.84, 1.43]	0.50	2%
Sucralfate	2	1.62 [0.66, 3.94]	0.29	78%
Duration of administration				
≤7 days	4	1.16 [0.84, 1.62]	0.37	0%
≤14 days	7	1.09 [0.85, 1.40]	0.49	0%
≤90 days	12	1.04 [0.94, 1.14]	0.47	5%
>90 days	4	1.34 [0.86, 2.09]	0.20	58%
Method of administration				
Intravenous	10	1.01 [0.93, 1.09]	0.86	0%
Oral (including PEG or NG tube)	7	1.27 [0.88, 1.85]	0.20	39%
Times of administration during 24-h period				
Once	14	1.07 [0.95, 1.21]	0.25	23%
Twice	5	1.07 [0.87, 1.31]	0.51	0%

No, number; RR, relative risk; CI, confidence interval; y, year; d, day; CVD, cardiovascular disease; PPI, proton pump inhibitor; ICU, intensive care unit; NG, nasogastric; PEG, percutaneous endoscopic gastrostomy; VAP, ventilator-associated pneumonia; HAP, hospital-acquired pneumonia.

Regarding participant comorbidities, pooled statistical results showed no statistically significant difference in pneumonia incidence between the experimental and control groups for patients with nerve injury (RR = 1.30, p = 0.14) or for patients with CVD (RR = 1.02, p = 0.77). Notably, significant heterogeneity was absent (*I* ^*2*^ = 0%). Moreover, classification based on ICU admission status revealed no significant differences in pneumonia morbidity between the PPI-exposed and non-PPI-exposed groups, regardless of whether participants were in the ICUs (RR = 1.05, p = 0.29) or out of the ICUs (RR = 1.28, p = 0.14). More detailed information is provided in Table 3.

**FIGURE 3:**
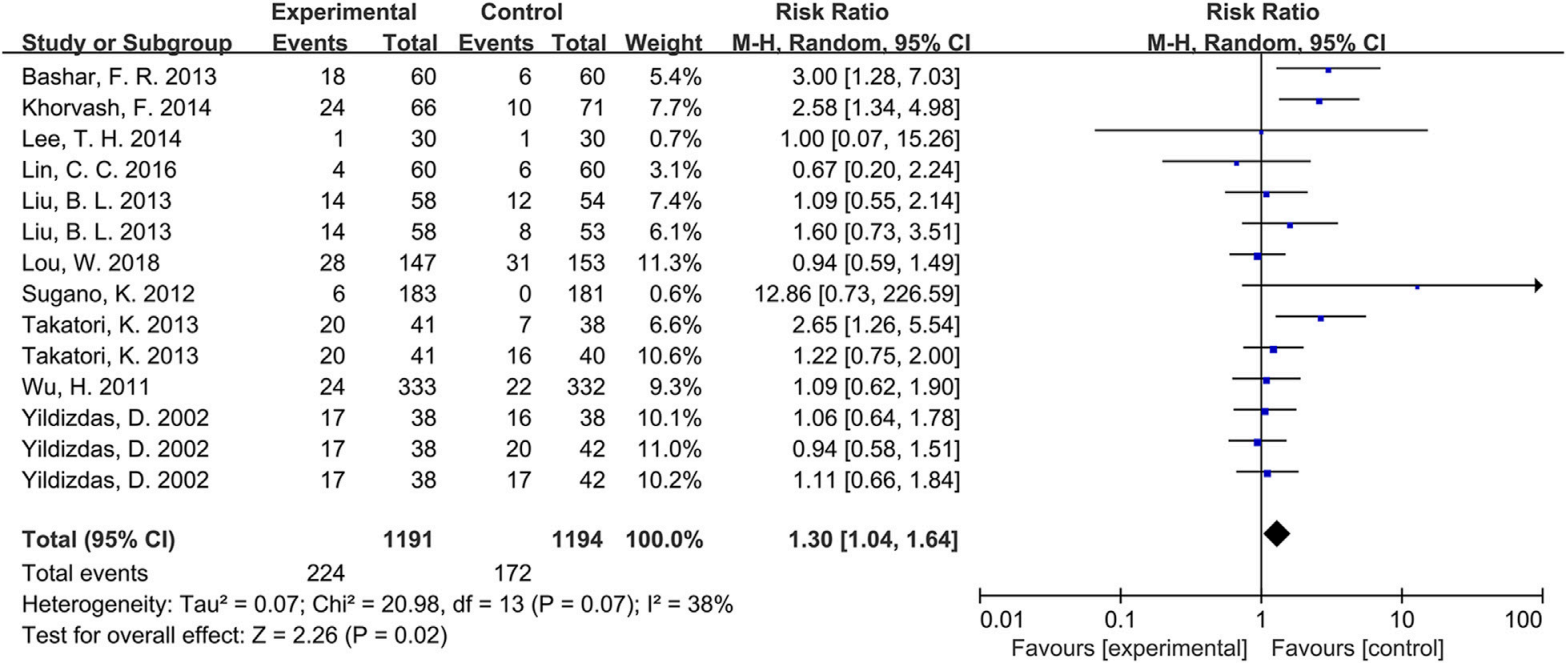
Forest plot of the effect of PPI use on the incidence of pneumonia in Asian population.

Concerning geographic origin, among participants from Asia (involving China, Japan, Iran, and Egypt), the intervention group exhibited a higher incidence of pneumonia compared to the control group (RR = 1.30, p = 0.02, Figure 3), with moderate heterogeneity (*I* ^*2*^ = 38%). Conversely, pooled results from two studies (Krag et al., 2018; Oliynyk, 2021) involving European participants indicated no significant differences in pneumonia incidence between groups (RR = 1.04, p = 0.67). Similarly, for participants from America, two studies (Holbrook et al., 2012; Somberg et al., 2008) demonstrated no significant differences in pneumonia incidence between both groups (RR = 1.02, p = 0.95). Further details are displayed in Table 3.

**FIGURE 4:**
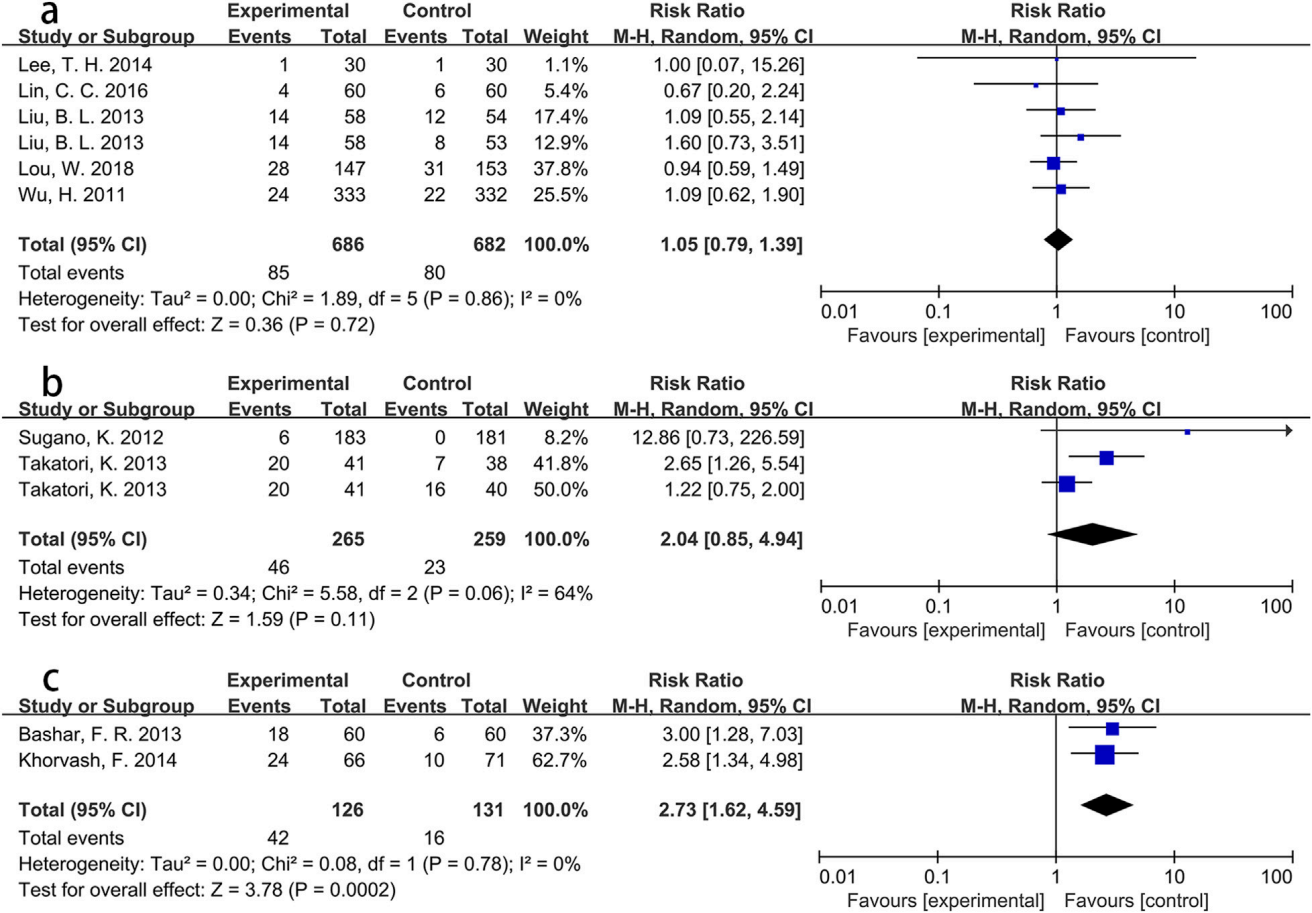
Forest plot of the effect of PPI use on the incidence of pneumonia in specific Asian countries (**(a)** China; **(b)** Japan; **(c)** Iran).

**FIGURE 5:**
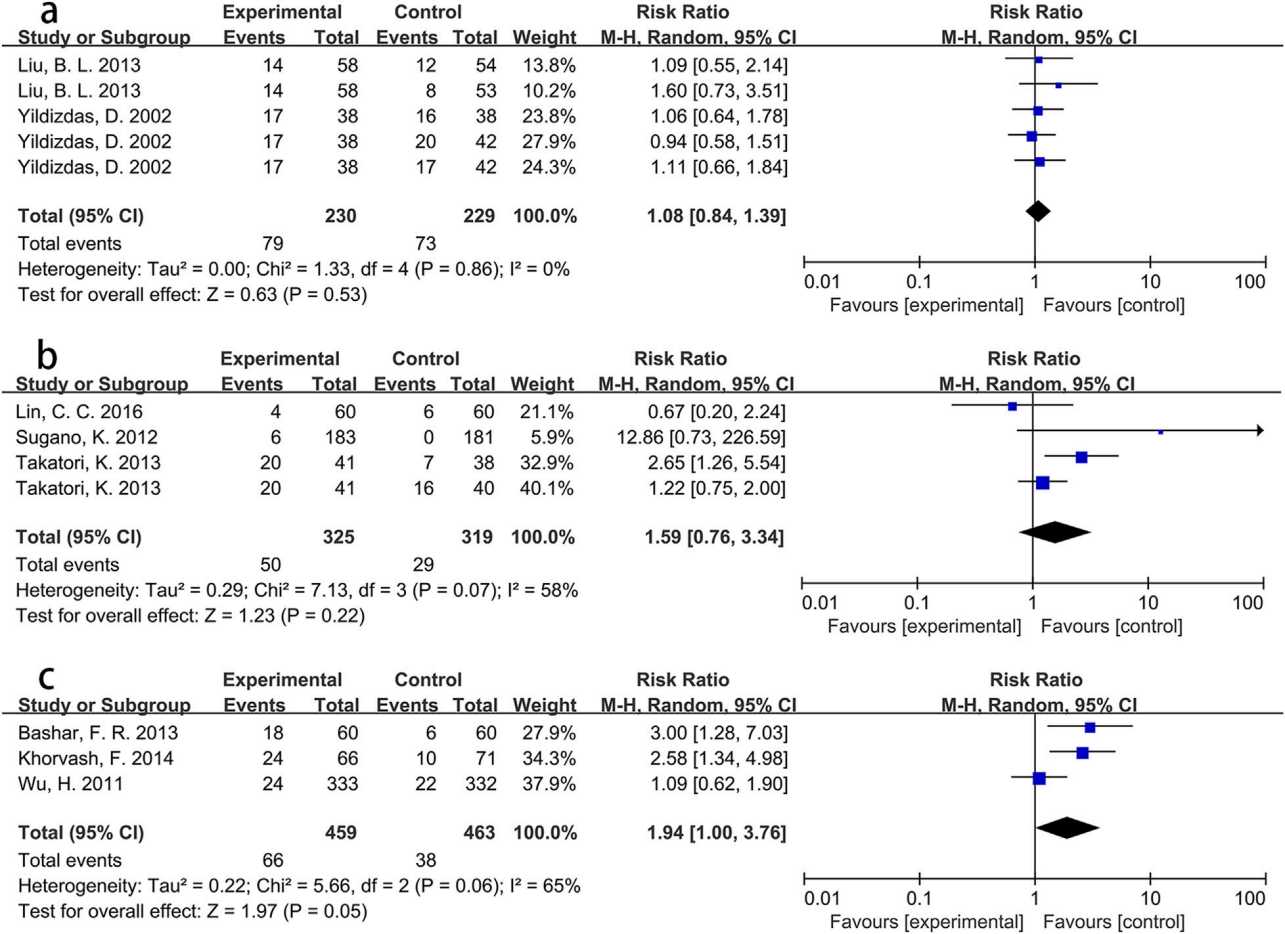
Forest plot of the effect of PPI use on the incidence of pneumonia in Asian population using different PPIs (**(a)** omeprazole; **(b)** lansoprazole; **(c)** pantoprazole).

To explore these findings further, additional subgroup analyses were performed based on specific Asian countries (involving China, Japan and Iran) and PPI types. For participants from Iran, the intervention group showed a significantly greater incidence of pneumonia than the control group (RR = 2.73, p < 0.001), with low heterogeneity (*I* ^*2*^ = 0%). However, no significant differences were found for participants from China (RR = 1.05, p = 0.72) or Japan (RR = 2.04, p = 0.11). Detailed information is presented in Figure 4. Pooled results from three Asian studies (Bashar et al., 2013; Khorvash et al., 2014; Wu et al., 2011) administering pantoprazole indicated a higher morbidity of pneumonia in participants receiving pantoprazole compared to controls (RR = 1.94, p = 0.05), albeit with significant heterogeneity (*I* ^*2*^ = 65%). Conversely, participants using lansoprazole (RR = 1.59, p = 0.22) or omeprazole (RR = 1.08, p = 0.53) showed no significant between-group differences in pneumonia incidence. Further details are shown in Figure 5.

With respect to pneumonia type as an adverse outcome, the RR for VAP incidence was 1.17, showing no significant difference between groups (p = 0.11). Notably, the intervention group exhibited a higher morbidity of HAP than the control group, although this difference did not reach statistical significance (RR = 1.12, 95% CI: 1.00-1.26, p = 0.06). More detailed information is presented in Table 3.

When analyzing by PPI types specifically, no significant differences in pneumonia rates were observed between the intervention and control groups for participants using omeprazole, lansoprazole, pantoprazole, or esomeprazole (all p > 0.1). Analogously, no significant differences in pneumonia morbidity were found between groups based on the control measure employed (placebo/no drug, H2 receptor antagonist, or sucralfate; all p > 0.1). Further details are displayed in Table 3.

Subgroup analyses based on administration duration (≤7 days, ≤14 days, ≤90 days, >90 days), dosing frequency within a 24-h period (once, twice), and route of administration (intravenous, oral including PEG or NG tube) all manifested no significant differences in pneumonia rates between the two groups (all p > 0.1). More detailed information is presented in Table 3.

### Publication bias and sensitivity analysis

To evaluate publication bias regarding the association between PPI exposure and pneumonia risk, a funnel plot was employed and Begg’s test was performed for statistical verification. The funnel plot demonstrated approximate symmetry (Supplementary Figure S3), and Begg’s test did not indicate significant bias (p = 0.302; Supplementary Figure S4), collectively suggesting the absence of substantial publication bias. Additionally, sensitivity analysis, which sequentially excluded individual studies, confirmed that the statistical results remained stable (Supplementary Figure S5).

## Discussion

This meta-analysis of 20 RCTs (Abu El-Ella et al., 2022; Alhazzani et al., 2017; Bashar et al., 2013; Cook et al., 2024; Holbrook et al., 2012; Khorvash et al., 2014; Krag et al., 2018; Lee et al., 2014; Lin et al., 2016; Liu et al., 2013; Lou et al., 2018; Moayyedi et al., 2019; Oliynyk, 2021; Orenstein et al., 2009; Selvanderan et al., 2016; Somberg et al., 2008; Sugano et al., 2012; Takatori et al., 2013; Wu et al., 2011; Yildizdas et al., 2002) observed a potential increased risk of pneumonia associated with PPI use. This association was particularly evident for HAP and within Asian populations (involving China, Japan, Iran and Egypt), with pronounced effects observed specifically in Iran and among Asian users of pantoprazole. Conversely, no significant association between PPI use and pneumonia risk was identified for participants from Europe or America, for VAP, for patients regardless of ICU admission status, or across subgroup analyses based on age, comorbidities, control group types (placebo/no drug, H2 receptor antagonist, sucralfate), or PPI administration regimen (including duration, methods of administration and dosing frequency within 24 h).

A nested case-control study demonstrated that PPI users had approximately fourfold higher odds of developing pneumonia compared to non-users (Sarkar et al., 2008). Gastric acid serves as the primary gastrointestinal defense barrier, performing essential physiological functions including digestion and pathogen suppression by inhibiting microbial colonization and proliferation. Critically, PPIs suppress gastric acid secretion through irreversible covalent binding to H^+^/K^+^-ATPase (Shin and Sachs, 2008). This inhibition may compromise gastric acid’s natural protective role, potentially increasing pneumonia susceptibility via several mechanisms. (1) Studies have confirmed gastric pH < 2 effectively limits microbial colonization (Savarino et al., 2009). PPIs elevate gastric pH > 4 for prolonged periods (Hunt et al., 2005), promoting gastric bacterial overgrowth and delayed emptying. These factors increase aspiration risk, facilitating pulmonary pathogen exposure. (2) PPI-mediated acid suppression induces intestinal microbial dysbiosis (Hojo et al., 2018), enriching opportunistic pathogens. This disturbance may indirectly alter respiratory tract microenvironments through gut-lung axis interactions (Ye et al., 2025), elevating pneumonia risk. (3) Animal studies have indicated that PPI-induced gastric pH elevation can compromise gastrointestinal tight junctions, increasing epithelial permeability (Nighot et al., 2023). This barrier dysfunction may promote intestinal microbiota translocation into systemic circulation, potentially triggering pulmonary inflammatory responses.

Beyond compromising gastric acid barriers, PPIs may promote immune dysregulation through multiple pathways. (1) PPIs inhibit caspase-3 and caspase-8, inducing apoptosis in polymorphonuclear leukocytes (Capodicasa et al., 2008). Concurrently, they disrupt chemotactic migration and leukocyte recruitment via altered signal transduction and gene expression (Fowler et al., 2024), thereby weakening phagocytic and bactericidal capabilities. (2) By inhibiting H^+^/K^+^-ATPase in neutrophils and disrupting cation flux across cell membranes, PPIs reduce intracellular calcium availability (Martins de Oliveira et al., 2007). This impairs lysosomal phagocytic function (Aybay et al., 1995), which is a critical process for pathogenic bacterial elimination. (3) *In vitro* evidence has indicated PPIs interact with natural killer (NK) cells, significantly reducing their cytotoxic function through a potential drug-immune system (Aybay et al., 1995; Capodicasa et al., 1999). (4) Vitamin B12 plays a critical role in anti-inflammatory processes and immune regulation. A recent study has demonstrated that prolonged PPI use induces vitamin B12 deficiency in Zollinger-Ellison Syndrome patients (Ito et al., 2024). Furthermore, existing research confirms that reduced serum vitamin B12 levels correlate with adverse clinical outcomes in COVID-19 (Shakeri et al., 2022). Collectively, these mechanisms indicate PPIs may systemically compromise immune defenses, potentially diminishing pulmonary antimicrobial responses and elevating pneumonia susceptibility.

Significantly, respiratory tract mucus normally maintains a weakly acidic environment that inhibits pathogenic bacterial proliferation (Fischer and Widdicombe, 2006). Physiologically, H^+^/K^+^-ATPase expression occurs not only in gastric parietal cells but also in respiratory tract glandular epithelia (Dębczyński et al., 2023). Therefore, PPI administration may plausibly neutralize respiratory mucus pH, creating a microenvironment favorable for colonization by pneumonia-causing pathogens such as *Streptococcus* pneumoniae (Kadioglu et al., 2008) and *Staphylococcus aureus* (Wang et al., 2024b). Furthermore, such pH alterations may impair ciliary beat frequency and compromise mucociliary clearance efficiency (Xie et al., 2020).

Regarding pneumonia subtypes, current evidence confirms PPI use elevates pneumonia risk. A retrospective cohort study of 307,622 Chinese hospital admissions demonstrated that prophylactic PPI administration increased HAP incidence among glucocorticoid-treated patients (Mao and Yang, 2022). This was consistent with our findings. Possible reasons beyond the described mechanisms potentially include: (1) hospitalized patients have compromised baseline health and diminished antimicrobial defenses; (2) the ubiquitous presence of pathogenic bacteria in hospital settings, coupled with PPI-induced disruption of gastric acid barriers, respiratory tract microenvironment alterations and immune compromise, synergistically elevates pneumonia risk. Notably, this association was not observed for VAP, contradicting expected mechanisms. This discrepancy may stem from: (1) VAP has stronger association with antibiotic overuse and multidrug-resistant pathogens in mechanically ventilated patients (Čiginskienė et al., 2019); (2) potential statistical limitations from insufficient VAP subgroup sample size.

In this meta-analysis, the significantly higher incidence of pneumonia among Asian (involving China, Japan, Iran and Egypt) PPI users merits attention. This disparity may be attributable to two interrelated factors. (1) *H. pylori* infection prevalence is substantially higher in Asian populations than in Western counterparts (Hooi et al., 2017). This pathogen induces chronic gastritis and intestinal metaplasia, impairing gastric acid barrier function and promoting pathogenic microbial translocation (Čiginskienė et al., 2019). This baseline vulnerability likely potentiates PPI-associated pneumonia risk in Asian populations. (2) Asians exhibit higher frequencies of the CYP2C19 poor metabolizer phenotype (Zhou and Lauschke, 2022). This genetic profile elevates plasma concentrations of CYP2C19-metabolized PPIs (Fu et al., 2021), resulting in more potent and prolonged acid suppression. Such exaggerated pharmacodynamic effects may further destabilize gastric antibacterial defenses, facilitating pathogenic bacterial overgrowth.

However, this PPI-associated pneumonia risk trend was not observed in European and American countries, potentially attributable to the following. (1) Higher prevalence of the CYP2C19 rapid metabolizer phenotype accelerates PPI clearance (Zhou and Lauschke, 2022), reducing systemic exposure and potentially mitigating adverse effects. (2) Western populations typically consume high-protein diets, which stimulate gastrin secretion and enhance gastric acid production (Coate et al., 2014). This physiological response may partially offset PPI-mediated acid suppression. (3) The relatively small sample size of European/American participants across included studies reduced statistical power for regional subgroup comparisons.

Notably, further subgroup analysis of Asian participants (involving China, Japan, Iran and Egypt) demonstrated significantly elevated pneumonia risk among pantoprazole users. Potential explanatory mechanisms include: (1) pantoprazole exhibits reduced CYP2C19 binding affinity, resulting in decreased metabolic dependence on polymorphic variants of this enzyme (Ananthathandavan and Narayanasamy, 2025; Zhao et al., 2022); (2) the biphasic metabolic pathway characteristic of pantoprazole increases its bioavailability in humans (Cho et al., 2024). Additionally, among subjects from Iran in Asia, PPI use increased pneumonia risk. This phenomenon may relate to monotonous diet structure, deficient hygiene conditions, and poor nutritional status. However, these further subgroup analyses of the Iranian population and the pantoprazole users in Asia were derived from few studies with small sample sizes, which potentially exaggerated the true effect sizes and increased the risk of false-positive findings.

To date, a previous meta-analysis incorporating case-control and cohort studies demonstrated that PPI use increased the risk of community-acquired pneumonia (Xun et al., 2022). However, the inclusion of observational studies introduced bias, potentially compromising result reliability. Notably, a meta-analysis restricted to RCTs indicated no overall effect of PPI use on pneumonia risk (Wang et al., 2024a), but it lacked detailed subgroup analyses. Compared with the existing meta-analyses, the present study offers a more robust evaluation of the PPI-pneumonia link by integrating the gold-standard design of high-quality RCTs with extensive, pre-specified subgroup analyses based on age, comorbidities, geographic region, pneumonia classification, and PPI administration protocols.

Nevertheless, several limitations merit consideration. First, the inability to control for key VAP rick factors such as invasive interventions, oral care, 45-degree head of the bed, length of stay in intubation, reduced the credibility of the results. Second, the restricted number of included studies precluded additional subgroup analyses by factors such as sex, body mass index, or more types of comorbidities. Third, significant heterogeneity was observed, attributable to the variations in baseline health status of participants, PPI dosing regimens and clinical settings. Finally, the inclusion of small sample studies and the studies published only in English potentially affected the reliability of the results.

This study provides crucial, population-specific insights for PPI prescribing by revealing a spectrum of pneumonia risk in Asians (involving China, Japan, Iran and Egypt), with an overall increase of 4%–64%, a sharper rise of 62%–359% in Iran, and the highest risk peaking at 276% among pantoprazole users in Asia. In clinical practice, when PPI for patients in these high-risk groups, particularly those with additional risk factors for pneumonia, such as underlying respiratory conditions or immunocompromised status, clinicians should conduct a thorough benefit-risk evaluation, actively manage modifiable risk factors and periodic re-evaluation of the ongoing indication for therapy, including a trial of step-down therapy where appropriate. When acid suppression is necessary in Asian populations, pantoprazole should be used with particular caution, and alternative antisecretory agents should be considered if necessary. To guide more precise clinical decision, large-scale, prospective and multicenter RCTs conducted in diverse Asian populations are warranted to definitively confirm these subgroup findings.

## Conclusion

This meta-analysis suggests PPI use may elevate pneumonia risk in the general population, particularly among Asian (involving China, Japan, Iran and Egypt) subgroups (notably Iranian populations and Asian pantoprazole users) and for HAP, whereas no significant association emerged in European/American populations, VAP, patients regardless of ICU admission status, or subgroup analyses of age, comorbidities, control group type (placebo/no drug, H2 receptor antagonist, sucralfate) or PPI administration regimen (including duration, methods of administration and dosing frequency within 24 h).

## Data Availability

The original contributions presented in the study are included in the article/Supplementary Material, further inquiries can be directed to the corresponding author.

## Abbreviations

PPI, Proton Pump Inhibitor; RCTs, Randomized Controlled Trials; PEG, Percutaneous Endoscopic Gastrostomy; NG, Nasogastric; VAP, Ventilator-associated Pneumonia; HAP, Hospital-acquired Pneumonia; RR, Relative Risk; CI, Confidence Interval; CVD, Cardiovascular Disease; ICU, Intensive care unit; NK, Natural Killer; No, Number; PICU, Pediatric Intensive Care Unit; ICH, Intracranial Hemorrhage; UGI, Upper Gastrointestinal; GI, Gastrointestinal; NSAID, Nonsteroidal Anti-inflammatory Drug; ACS, Acute Coronary Syndromes; AP, Aspiration Pneumonia.
