# Supplementary figures and images for: The impact of proton pump inhibitor exposure on pneumonia: an updated meta-analysis based on randomized controlled trials
Source: Front Pharmacol. 2025 Oct 28;16:1713256. doi: 10.3389/fphar.2025.1713256 (PMC12602524; doi:10.3389/fphar.2025.1713256)

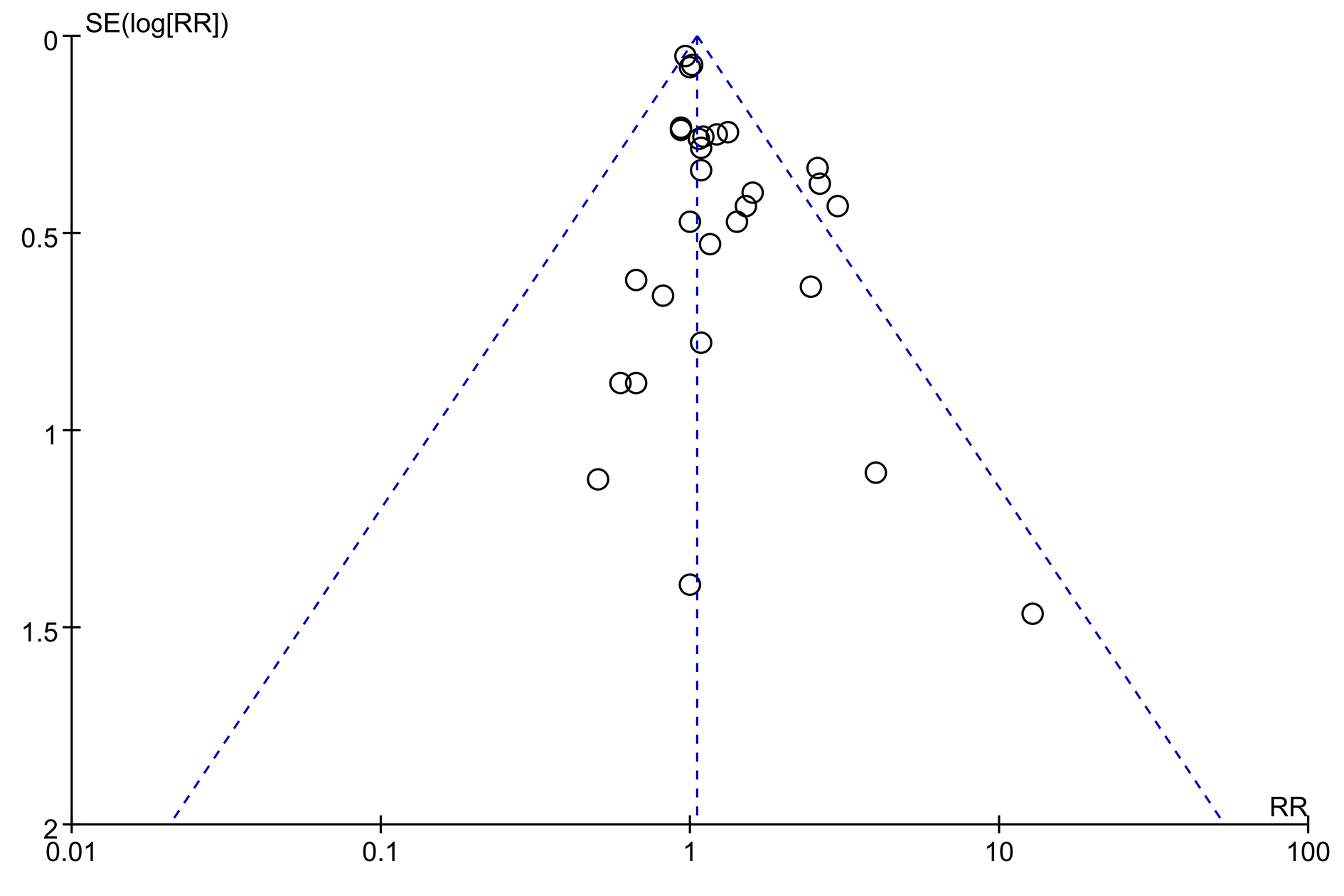

Supplement: Supplementary file 1 [file Image3.tif]

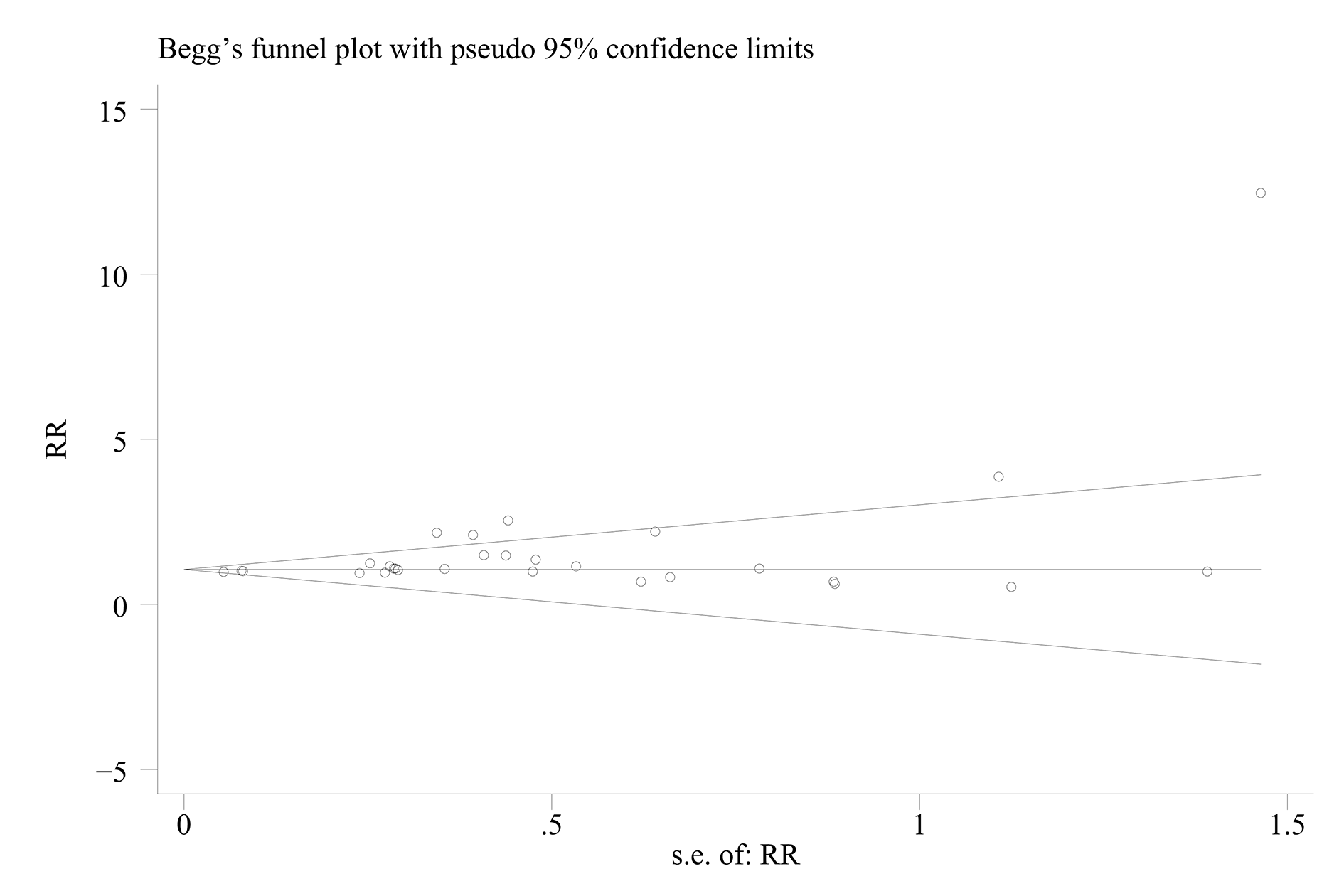

Supplement: Supplementary file 2 [file Image4.tif]

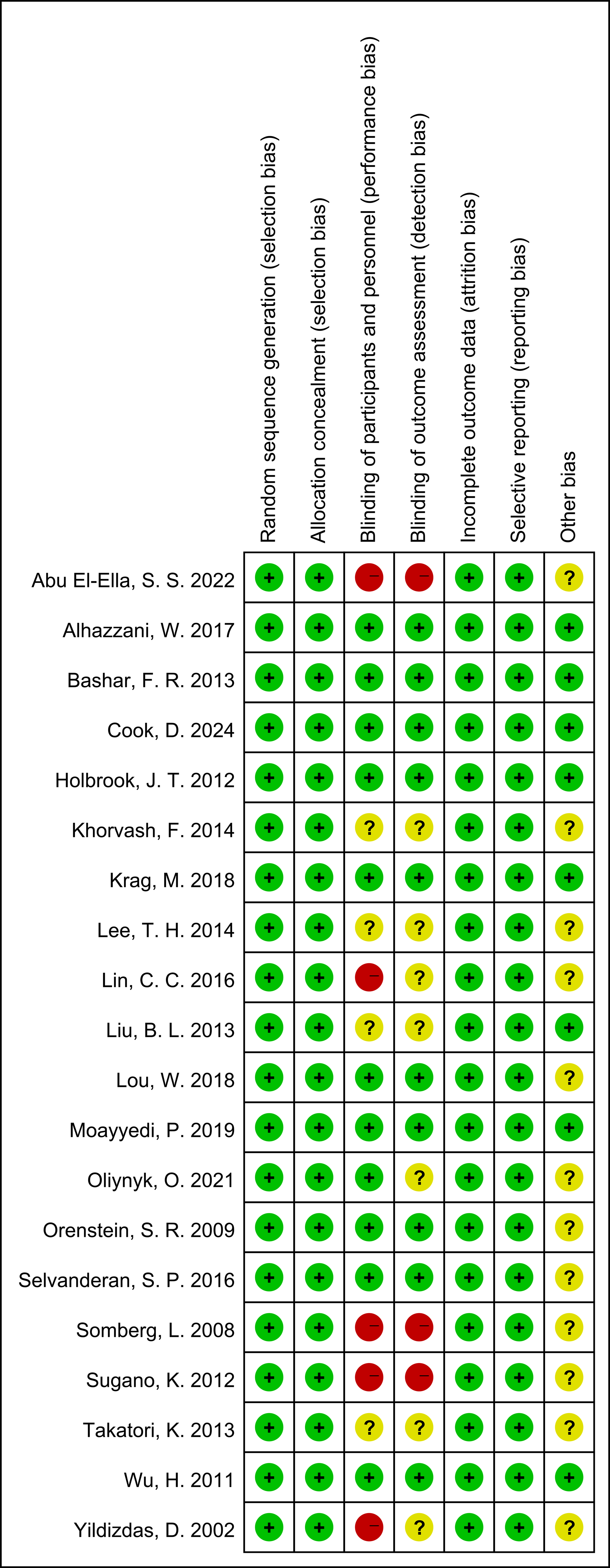

Supplement: Supplementary file 3 [file Image2.tif]

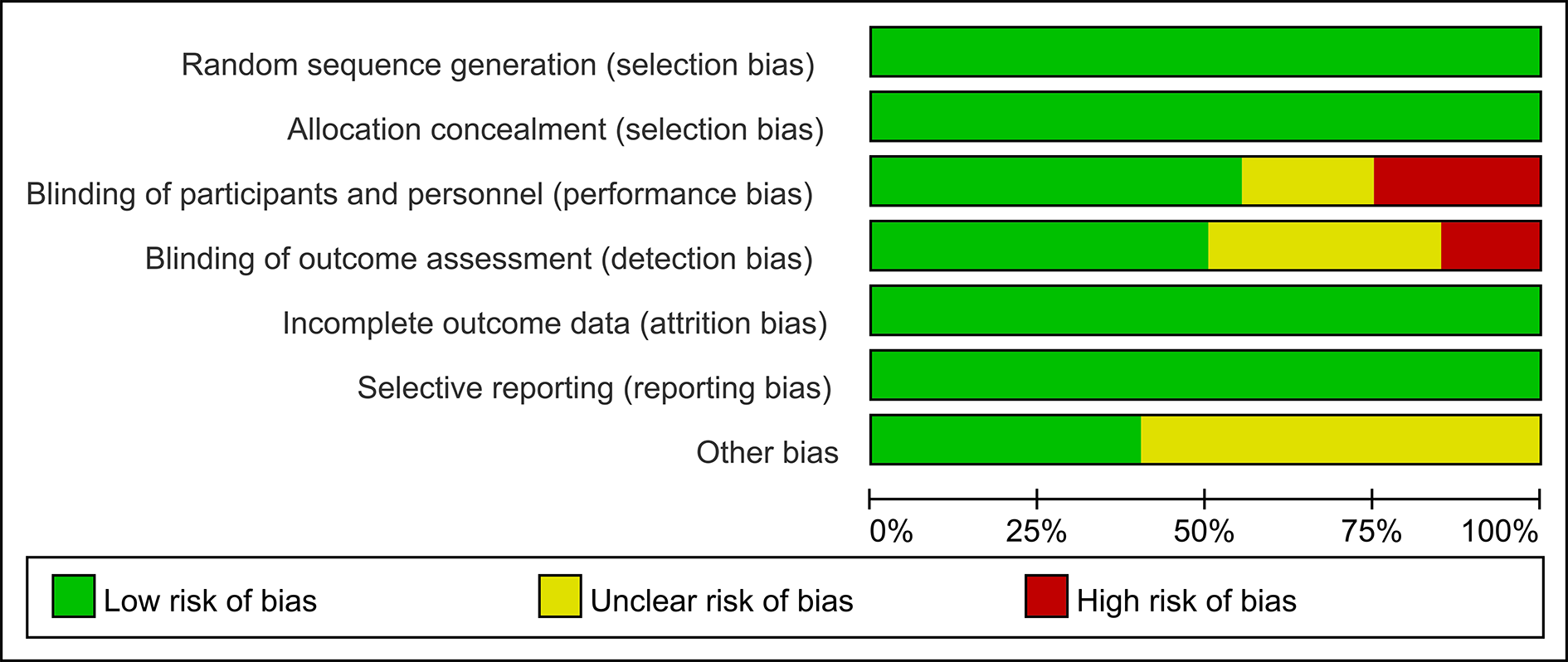

Supplement: Supplementary file 4 [file Image1.tif]

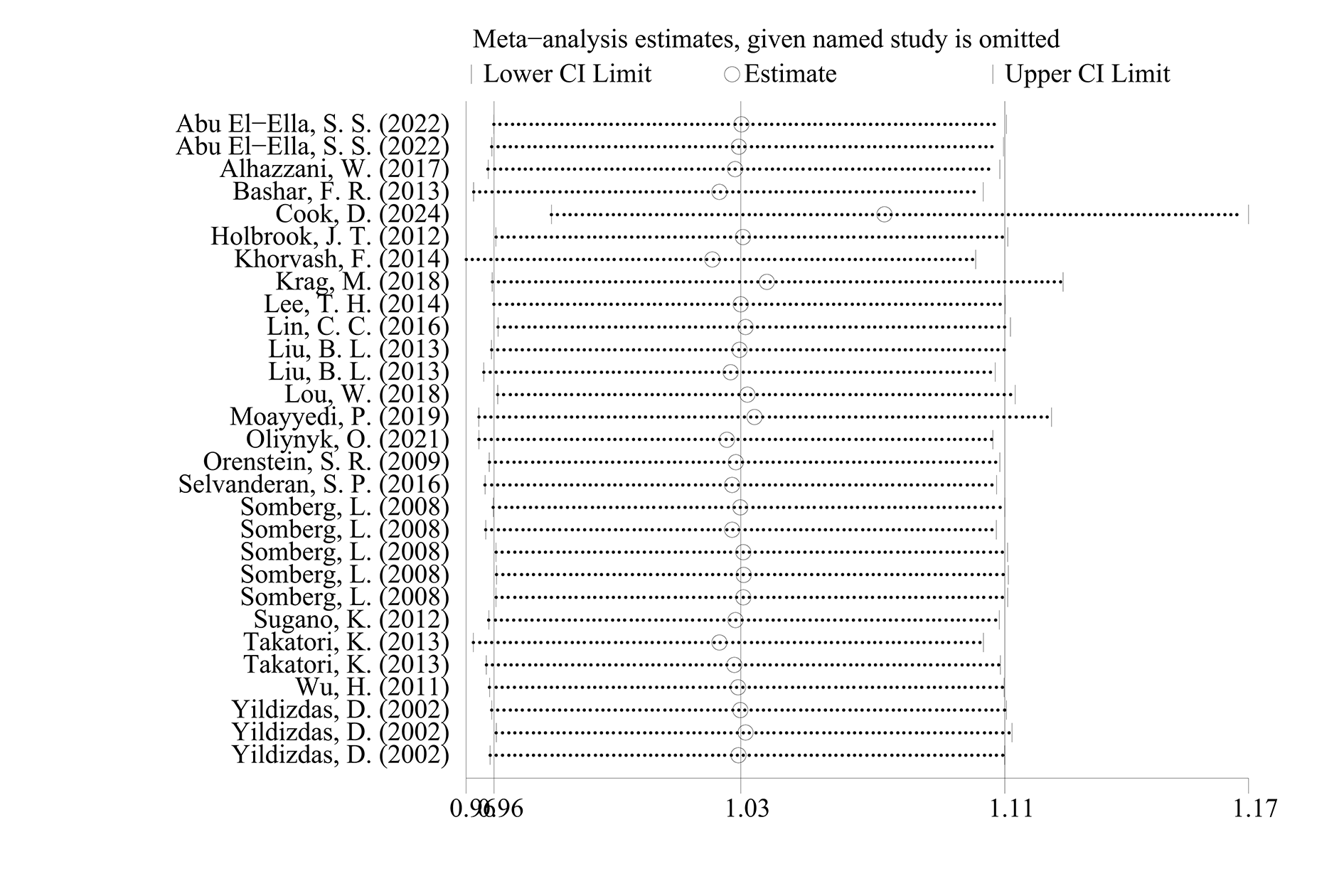

Supplement: Supplementary file 5 [file Image5.tif]
